# The Intranasal Administration of Semaphorin 3A Inhibitor in a Mouse Model of Olfactory Disorder

**DOI:** 10.7759/cureus.92587

**Published:** 2025-09-17

**Authors:** Aya Murai, Minori Noda, Aiko Shimizu, Junko Takahara, Seiichiro Makihara, Mizuo Ando

**Affiliations:** 1 Otolaryngology - Head and Neck Surgery, Okayama University Hospital, Okayama, JPN; 2 Division of Technical Support for Medical Science, Department of Comprehensive Technical Solutions, Okayama University, Okayama, JPN; 3 Otolaryngology - Head and Neck Surgery, Okayama University, Okayama, JPN

**Keywords:** axon growth, intranasal administration, olfactory disorder, olfactory sensory neurons, semaphorin3a

## Abstract

This study investigated the effects of intranasal administration of a semaphorin 3A inhibitor (Sema3A-I) in a mouse model of olfactory disorder, where olfactory sensory neuron (OSN) axons had been severely damaged. We performed axotomy (transection of OSN axons) of the OSNs in mice and administered Sema3A‑I intranasally to seven mice and saline to another seven mice. Following treatment, we assessed the thickness of the olfactory epithelium and the regeneration ratio of OSN axons. Intranasal administration of Sema3A-I did not significantly promote OSN regeneration, axonal outgrowth, or improve axonal projection compared to saline administration. Although Sema3A-I administration showed some promotion of axonal outgrowth, the difference was not statistically significant. Continuous subcutaneous administration of Sema3A-I in rats after axotomy promotes OSN regeneration and axonal outgrowth. Given that intranasal administration is minimally invasive, we believe that it may still be a feasible route when combined with additional treatment strategies. Further investigation into administration methods and therapeutic combinations is warranted.

## Introduction

Olfactory disorders significantly impair quality of life and are often caused by the disruption of the olfactory nerve pathway. Odorants entering the nasal cavity bind to receptors on olfactory sensory neurons (OSNs) located in the olfactory epithelium (OE). The axons of OSNs that express the same olfactory receptor converge into bundles and transmit signals to the olfactory bulb (OB), the primary center of olfactory processing. In the OB, OSNs form synapses with secondary neurons, which then relay information to higher olfactory centers, such as the olfactory cortex, hippocampus, and orbitofrontal cortex. During development, axon guidance cues expressed in the OB help form a neural circuit known as the glomerular map, which is preserved throughout life. 

OSNs possess three distinctive characteristics not seen in other sensory neurons. First, there is a one-to-one relationship in which one olfactory receptor is expressed per OSN [1,2]. Secondly, when OSNs project to the OB, the axons of OSNs expressing the same olfactory receptor converge to form a structure called the glomerulus in the OB [3]. The location of the glomeruli in the OB is called the glomerular map, which encodes olfactory information. Third, OSNs are continuously turned over throughout an organism’s lifetime. The glomerular map is formed during development by the action of axon guidance cues [3,4,5,6,7]. This map is maintained throughout life because the interrelationship between old and newly generated axons helps preserve the glomerular map [8]. However, if too many OSNs are damaged simultaneously, the regenerated OSNs may fail to reconstruct the correct neural circuitry in the OB [9]. In addition, while normal OSN axons project posteriorly within the OB, OSNs that regenerate after axotomy in mice often project anteriorly instead [9,10].

While many olfactory disorders are curable, some are resistant to therapy. Dysosmia refers to qualitative alterations or distortions in smell perception. Parosmia is a form of dysosmia characterized by the perception of an unpleasant odor triggered by a specific environmental stimulus, making it a distressing condition often associated with foul smells. Parosmia is frequently caused by a single event that leads to widespread damage of OSNs, such as viral infections or trauma [11,12]. In a previous study, OSNs that recovered after axotomy showed impaired neuronal axon outgrowth and were spatially found to exhibit axon mistargeting. Molecularly, this damage is believed to cause parosmia [10]. We wondered whether semaphorin 3A inhibitor (Sema3A-I) could improve axonal outgrowth and correct axonal projections. We also considered the simplicity of the administration method.

In this study, we focused on the Sema3A-I, which promotes axonal outgrowth. In experiments where rats were treated with Sema3A-I after axotomy using a subcutaneous pump, recovery following OSN damage was accelerated [13]. However, continuous subcutaneous administration of Sema3A-I is invasive. Fortunately, OSNs are in contact with the external environment; therefore, intranasal administration is a viable pathway. We investigated the efficacy of intranasal Sema3A-I as a novel treatment for olfactory dysfunction by promoting OSN and axon outgrowth and improving axon mistargeting.

## Materials and methods

Animals

MOR29A/29B transgenic mice (with MOR29A and MOR29B olfactory receptors fluorescently labeled with ECFP and EYFP, respectively) were obtained from Nara Medical University under a Material Transfer Agreement with Okayama University [14]. This study was approved by the Institutional Animal Care and Use Committee and carried out according to the Okayama University Animal Experimentation Regulations.

Axotomy of OSNs

Axotomy [9] was performed as previously described, with some modifications. Male mice aged 8-12 weeks were anesthetized with ketamine (80 μg/g body weight) and xylazine (7 μg/g), followed by subcutaneous injection of dexamethasone (0.1 μg/g; Kyoritsu Seiyaku) and enrofloxacin (5 μg/g; Pfizer) to prevent brain edema and infection. A dental drill was used to thin the nasal bone at the OE-OB border (along the cribriform plate). OSN axons projecting to the dorsal OB were transected along the cribriform plate using a micro knife for ophthalmic surgery (Beaver Xstar Slit Knife 2.8 mm, 45° bevel up, Beaver-Visitec International, Waltham, MA). After axotomy, a coverslip with gentamycin was placed, and the skin flap was sutured. In most experiments, only OSN axons projecting to the right OB were transected. Mice were placed in single-housed cages and maintained for 42 days to allow for recovery.

Sema3A inhibition

**Figure 1:**
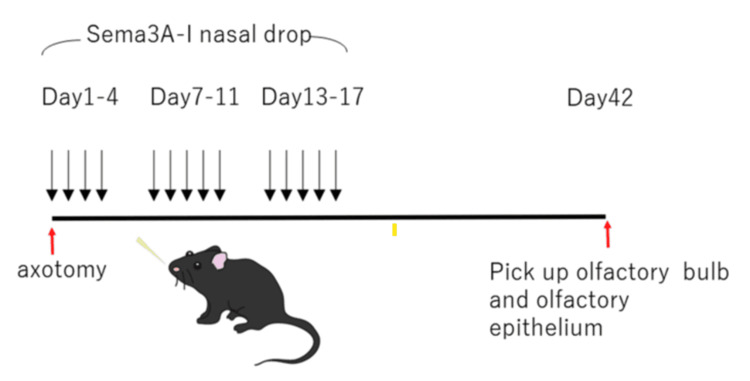
Intranasal Sema3A-I administration protocol after axotomy Axotomy was performed on the right side of the mice. Sema3A-I was administered intranasally once daily for three weeks starting after axotomy, following the described protocol. The OE and OB were observed on day 42 after axotomy Sema3A-I: semaphorin 3A inhibitor; OE: olfactory epithelium; OB: olfactory bulb

Sema3A inhibitor (1 μg/mL) was administered intranasally into the right nasal cavity in 20 μL doses for three weeks immediately after OSN damage. Sema3A-I (SM-345431) was provided by Sumitomo Pharma Co., Ltd. (Osaka, Japan) under a Material Transfer Agreement (Figure 1). The Sema3A-I and control groups included eight and four animals, respectively.

Immunohistochemistry of sections

Mice were deeply anesthetized with an overdose of ketamine (Pharma) by injection, followed by intracardiac perfusion with phosphate-buffered saline (PBS). Dissected OE and OB tissues were fixed in 4% paraformaldehyde (PFA)/PBS. The fixed samples were decalcified with 0.5 M Ethylenediaminetetraacetic acid for seven days and then processed into formalin-fixed, paraffin-embedded tissue blocks. Paraffin sections (4 μm thick) were mounted on glass slides. In the OE sections, the primary antibodies used were rabbit anti-keratin (Z0622, DAKO) at 1:100. The secondary antibody used was Alexa Fluor 568-conjugated donkey anti-rabbit IgG (A11045, Thermo Fisher Scientific, Waltham, MA), both at 1:200. DAPI (Thermo Fisher Scientific) was used at a 1:1000 dilution. In the OE sections, the primary antibodies used were rabbit anti-keratin (Z0622, DAKO) at 1:100.

In the OB section, for anti-neuropilin 1 (Nrp1) immunostaining, antigen retrieval was performed by treating the sections with 10 mm citrate buffer (pH 6.0) at 120 °C for 20 minutes. The sections were blocked with 10% normal donkey serum. The primary antibodies used were goat anti-OMP (019-22291, FUJIFILM Wako Pure Chemical Corporation, Hong Kong) at 1:1000 and rabbit anti-Nrp1 (ab81321, abcam) at 1:50. The secondary antibodies used were Alexa Fluor 488-conjugated donkey anti-goat IgG (A11055, Thermo Fisher Scientific) and Alexa Fluor 568-conjugated donkey anti-rabbit IgG (A11045, Thermo Fisher), both at 1:200. DAPI (Thermo Fisher Scientific) was used at 1:1000 dilution.

Observation of regenerated OSNs after damage

The OE and bulb of Sema3A-I-treated animals were observed 42 days after OSN damage. In previous studies, OE was confirmed to be regenerated by newly formed OSNs within 42 days post-axotomy; however, axonal projections to the paired OB glomeruli remained incorrect. Measurements of OSNs and basal cells in the OE were taken at five arbitrarily selected locations.

**Figure 2:**
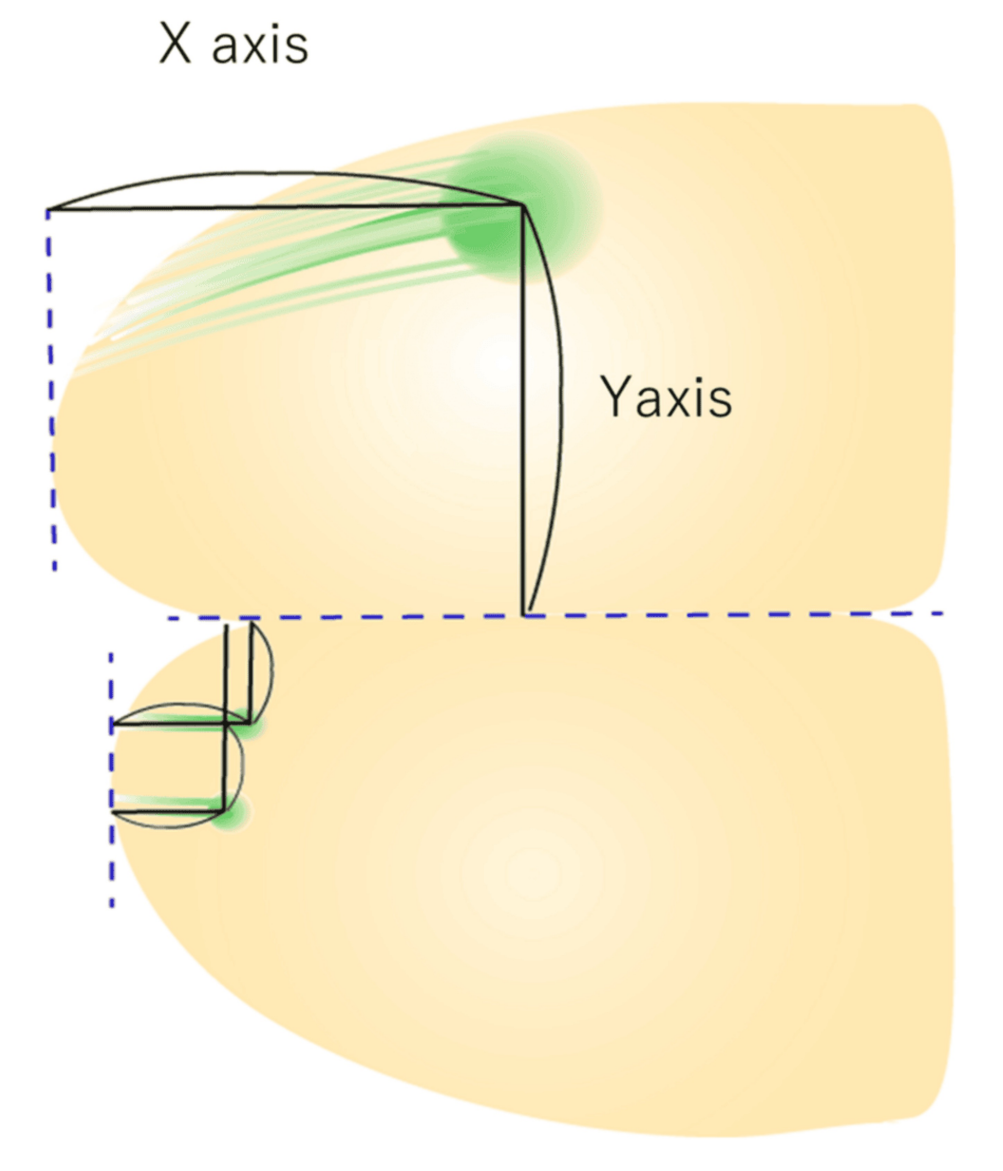
The lengths of regenerated axons were measured along the X axis and Y axis as shown in the figure Ratios relative to the control side were calculated

The spatial arrangement of glomeruli receiving input from newly formed OSNs was observed using a fluorescence microscope (OLYMPUS stereoscopic microscope SZX12, Central Research Laboratory, Okayama University Medical School). The lengths of regenerated axons were measured along the X and Y axes, and ratios were calculated in comparison with the control side (Figure 2).

Statistical analysis

Statistical analyses were performed using Prism 9. The thickness of the OE, the quantification of OSNs and basal cells in the OE, and the X- and Y-axis lengths of regenerated OSN axons were analyzed using the Mann-Whitney U test. Statistical significance was set at p<0.05.

## Results

Of the eight animals in the intranasal Sema3A-I group and eight in the control (intranasal saline) group, one animal died during the experiment. Sema3A-I was used at its dilution limit. Because the mice breathe exclusively through the nose, both the axotomy and intranasal administration were performed only on the right side to prevent asphyxia. To avoid respiratory distress, the maximum intranasal volume administered at a time was limited to 5 µL. Each subsequent 5 µL dose was given after a five-minute interval, totaling 20 µL per session.

**Figure 3:**
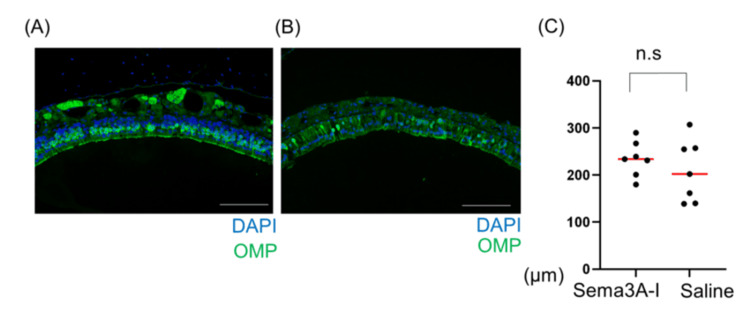
Recovery of OSNs 42 d after axotomy in OE Recovery of OSNs 42 d after axotomy intranasal administration of (A) Sema3A-I group and (B) Saline group in OE. Scale bar: 100 μｍ. (C) Assessment of OE thickness and regenerated axon outgrowth (p = 0.5350, Mann-Whitney U test) OSN: olfactory sensory neuron; OE: olfactory epithelium; Sema3A-I: semaphorin 3A inhibitor

The average thickness of the OE was measured at five arbitrary points in each group. No statistically significant differences were observed between the intranasal Sema3A-I and saline groups (Figure 3).

**Figure 4:**
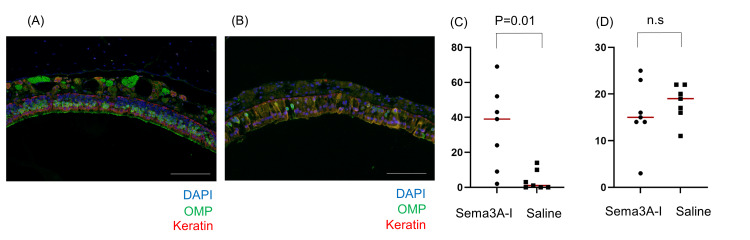
OSNs and basal cells in the OE Recovery of OSNs 42 days after axotomy intranasal administration of (A) Sema3A-I group and (B) Saline group in OE. Scale bar: 100 μｍ. (C) The quantification of OSNs in the OE (p = 0.0105, Mann-Whitney U test). (D) The quantification of basal cells in the OE (p = 0.4796, Mann-Whitney U test) OSN: olfactory sensory neuron; OE: olfactory epithelium; Sema3A-I: semaphorin 3A inhibitor

However, when we examined the recovery of the olfactory‐epithelium cells themselves, the Sema3A‑I group showed a greater number of olfactory sensory neurons compared to the control group. There was no statistically significant difference in the basal cells (Figure 4).

**Figure 5:**
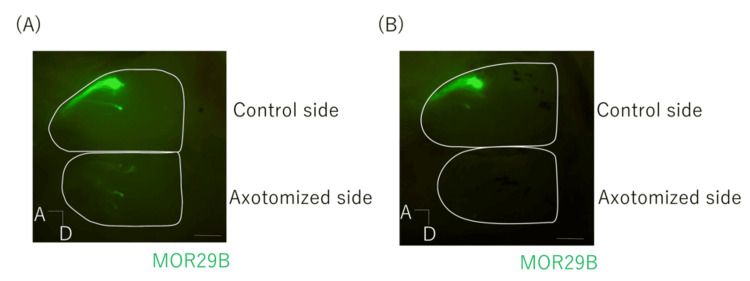
Recovery of OSN axons 42 days after axotomy intranasal administration of Sema-I MOR29B-expressing OSN axons projected to the OB Fluorescent green indicates the MOR29B olfactory receptor. Following axotomy, panels show the growth of OSN axons in the OB after intranasal administration of (A) Sema3A‑I and (B) saline. Scale bar 500 μｍ. A: anterior side; D: dorsal side OSN: olfactory sensory neuron; OB: olfactory bulb; Sema3A-I: semaphorin 3A inhibitor

MOR29B-expressing OSN axons projected to the dorsolateral region of the posterior OB; however, they did not return to their original projection sites in either the Sema3A-I or saline group. Axonal outgrowth in the OB was measured along the X- and Y-axes. While some regenerated axons showed enhanced outgrowth in the OB, others displayed no difference between the Sema3A-I and saline groups (Figure 5).

**Figure 6:**
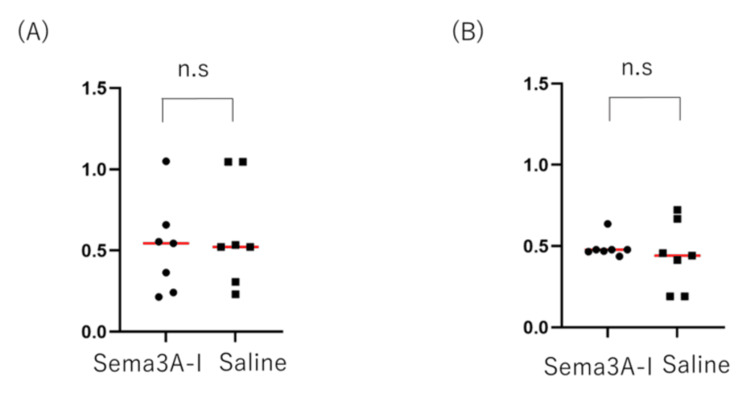
Length of regenerated OSN axons along the X axis and Y axis in OB Some regenerated axons appeared to grow along the anterior-posterior axis, but no significant difference was observed. (A) Measurement of the X-axis of OSN axons in the OB of mice; intranasal administration of Sema3A-I and saline following axotomy. p>0.9999 (Mann-Whitney U test. (B) Measurement of the Y-axis of OSN axons in the OB (p = 0.8048, Mann-Whitney U test) OSN: olfactory sensory neuron; OB: olfactory bulb; Sema3A-I: semaphorin 3A inhibitor

Although axonal extension tended to be greater in the experimental group, the difference was not statistically significant (Figure 6). No changes in projection patterns were observed in either the intranasal Sema3A-I or control group.

**Figure 7:**
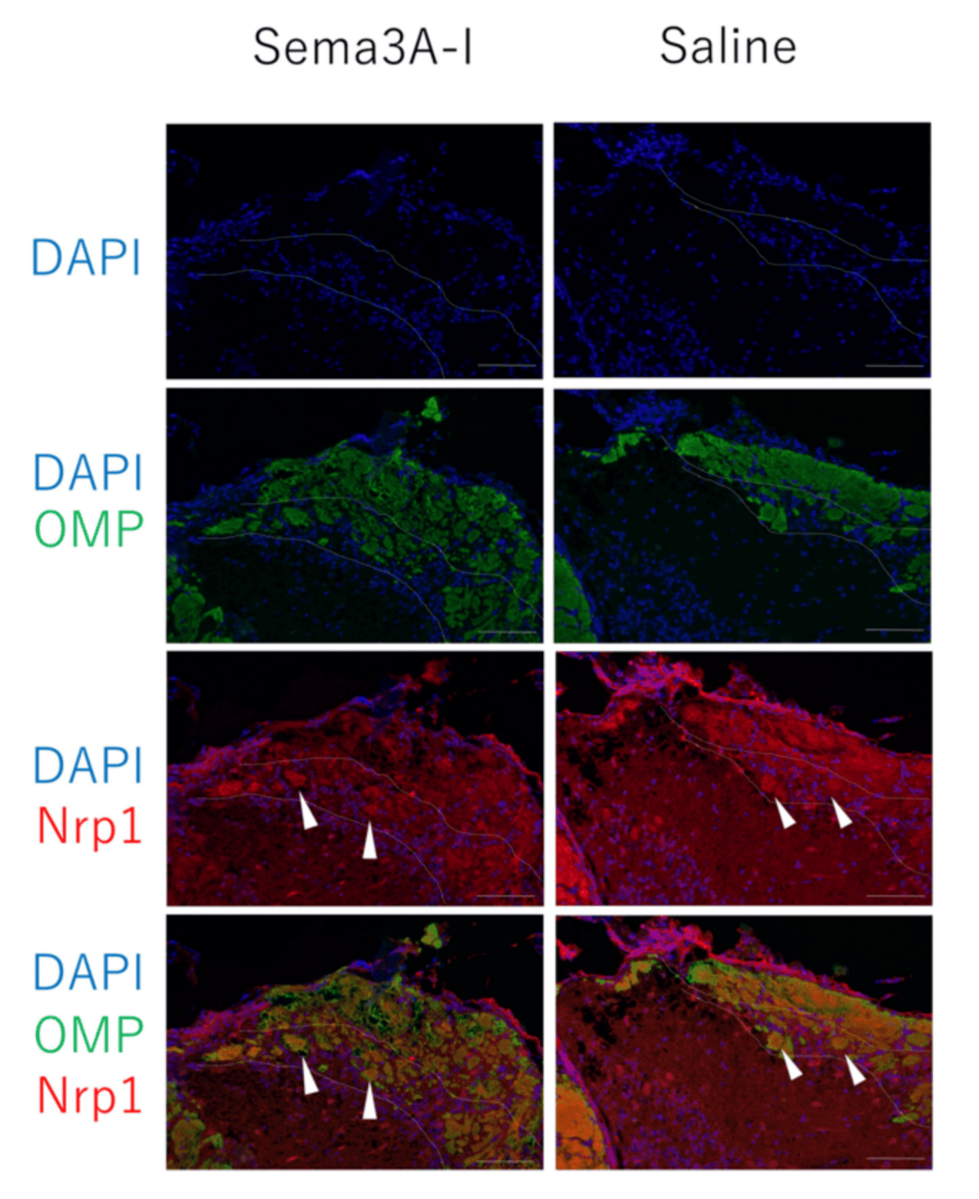
Appearance of Nrp1-positive axons in the OB after intranasal administration of Sema3A-I and saline In the control OB, Nrp1-positive axons were located in the superficial layer of the olfactory nerve layer and projected to the posterior OB. However, in both the intranasal Sema3A-I and saline groups after axotomy, Nrp1-positive axons were found in deeper regions of the olfactory nerve layer and invaded glomeruli in the anterior OB (arrowheads). Scale bar 100μm Nrp1: neuropilin 1; OB: olfactory bulb; Sema3A-I: semaphorin 3A inhibitor

Regenerated axons were also examined at the molecular level. Sema3A-Nrp1 signaling plays a role in axon-target interactions that guide the glomerular map in the OB during development. Under normal conditions, Nrp1-high OSN axons project to posterior glomeruli [10]. In the anterior OB, Nrp1-high axons are typically sorted into the superficial olfactory nerve layer. After axotomy, however, regenerated Nrp1-high OSN axons in both the intranasal Sema3A-I and saline groups were frequently found ectopically in the deep layer of the nerve and projected to glomeruli in the anterior OB. Intranasal administration of Sema3A-I did not correct the mistargeting of regenerated axons at the molecular level (Figure 7).

## Discussion

This study yields two key findings. First, intranasal administration of Sema3A‑I to OSNs promoted axonal outgrowth. Although the difference did not reach statistical significance, enhanced axonal growth was observed in a subset of OSNs. However, Sema3A‑I failed to improve axonal mistargeting. On the other hand, while Sema3A‑I did not affect the thickness of the OE, it may have contributed to the regeneration of OSNs themselves.

Olfactory disorders are common, not only in Japan but globally. Conductive olfactory disorders are often caused by rhinological conditions such as chronic sinusitis and allergic rhinitis, and are typically curable with therapies targeting these underlying causes. However, olfactory disorders resulting from damage to OSNs due to viral infections, trauma, drugs, or central nervous system diseases (e.g., Alzheimer's and Parkinson's diseases) are more difficult to treat and often incurable [14]. In this study, we focused on the axon guidance cue, Sema3A. During development, Sema3A is expressed in the OB, and its signaling with Nrp1 helps form the glomerular map-nrp1-high OSNs project to the posterior OB. After the critical period, if Sema3A is still expressed, Nrp1-high OSNs can project appropriately even following axotomy.

We previously administered Sema3A to the OB via slow-release beads after axotomy to determine if axonal targeting could be restored. However, this treatment inhibited axon outgrowth and failed to prevent mistargeting. Sema3A is also expressed during inflammation; it may act more as a repellent factor than a guidance cue after the critical developmental period. Therefore, we hypothesized that inhibiting Sema3A could promote axonal length and correct glomerular targeting in regenerated OSNs. In this study, we administered Sema3A-I intranasally and observed a trend toward increased axon outgrowth in the Sema3A-I group, though this was not statistically significant. These results align with previous studies, suggesting that Sema3A-I may positively influence OSN recovery in a subset of axons.

In this study, although the thickness of the OE did not change, the recovery of OSNs was observed following administration of Sema3A‑I. There are various cell types in the OE besides the OSNs, including supporting cells and Bowman's gland cells. In the present Sema3A‑I experiment, there was no significant difference in the thickness of the OE compared with the control group, and since the number of OSNs had increased, it is possible that the factors involved in neuronal outgrowth affect sensory cells specifically. Also, although not statistically significant, the impression was that the control group had more basal cells, which are undifferentiated olfactory cells. Considering that we are observing a recovery in progress, these observations support the possibility that Sema3A‑I promotes the recovery of OSNs. Alternatively, it may take time for the promotion of OSNs regeneration to be reflected in the axons. We therefore revised our discussion to suggest that a longer observation period might reveal greater axonal outgrowth.

However, unlike earlier reports, this study did not observe promotion of olfactory epithelial recovery. This discrepancy may be due to differences in the administration methods. In prior experiments, Sema3A-I was delivered continuously via subcutaneous pumps [15,16]. Clinically, traumatic olfactory dysfunction significantly impacts quality of life, but the need for intravascular procedures to deliver treatment presents challenges for both clinicians and patients. Fortunately, the OE is accessible through the nasal cavity, making intranasal drug administration a relatively simple and noninvasive option. Given that the axon outgrowth was observed to some extent, intranasal administration appears promising, but further investigation into optimal delivery methods is needed.

Furthermore, we hypothesize that the nasal instillation itself may have an effect as a nasal lavage. Nasal saline irrigation has been reported to improve endoscopic and subjective symptom scores following endoscopic sinus surgery for chronic rhinosinusitis [17,18].In this experiment, a total of 20 μL of solution was instilled into the mouse nasal cavity. Comparing the nasal cavity volumes of humans and mice purely [19,20], and considering that the mice underwent an artificial injury centered on the skull base by axotomy, we administered nasal instillation of Sema3A-I based on saline solution and saline as a control to the site of injury. This may have had some effect on the lavage of the wound site and thus, the axonal regeneration.

The results of this study highlight the importance of drug delivery systems in treating olfactory dysfunction. The discrepancy with previous studies suggests that refining the delivery method may significantly influence treatment efficacy. Nasal steroids have long been used to treat olfactory disorders. In one study comparing local and systemic steroid administration after the common cold, systemic steroids improved olfactory thresholds in some patients, highlighting how delivery routes can affect treatment outcomes. However, not all effective drugs or delivery methods are clinically viable. It is essential to weigh the risks of drug administration against the potential benefits.

Given the risks associated with systemic steroid use, neither systemic nor topical steroids are currently recommended in Japan for post-cold or post-traumatic olfactory disorders. While ease of administration is important, it is meaningless if the treatment is ineffective. Additionally, intranasal administration in mice has dosage limitations due to their nasal breathing. Only 5 µL can be administered at a time; exceeding this volume may cause choking. Respiratory deterioration - not adverse drug reactions - was believed to be responsible for the deaths observed in some mice. Since the drug was already administered at its dilution limit, higher concentrations are not feasible, and further studies are needed to explore more effective and practical delivery methods.

This study highlights the potential limitations of intranasal Sema3A-I administration. However, there are several constraints, including sample size and optimization of the dosage and methods. A previous study on Sema3A-I and rehabilitation in a rat model of spinal cord injury showed the compound’s effectiveness, especially when combined with rehabilitation, compared to either treatment alone [21]. The synergistic effects of promoting axonal growth and central stimulation through rehabilitation warrant further exploration. Olfactory training has been proposed as a rehabilitation method for olfactory dysfunction [22], and our department has also found it effective in a mouse model. Future studies should investigate the combined effects of pharmacological treatment and rehabilitation strategies.

## Conclusions

In this study, Sema3A‑I was administered intranasally in a mouse model of olfactory disorder. Although no statistically significant difference in axonal outgrowth was observed compared with the control group, there was an indication that it may have contributed to promoting the regeneration of OSNs. We anticipate that intranasal delivery of Sema3A‑I may offer a minimally invasive and effective therapeutic approach for treating refractory olfactory disorders.
